# Supplementary material for: Benchmarking orthology methods using phylogenetic patterns defined at the base of Eukaryotes
Source: Brief Bioinform. 2020 Sep 16;22(3):bbaa206. doi: 10.1093/bib/bbaa206 (PMC8138875; doi:10.1093/bib/bbaa206)

Benchmarking orthology methods using phylogenetic patterns defined at the base of Eukaryotes.

Eva S. Deutekom, Berend Snel, Teunis J.P. van Dam

Theoretical Biology and Bioinformatics | Institute of Biodynamics and Biocomplexity | Department of Biology | Faculty of Science | Utrecht University | Padualaan 8, 3584 CH Utrecht | The Netherlands

Supplementary Figures

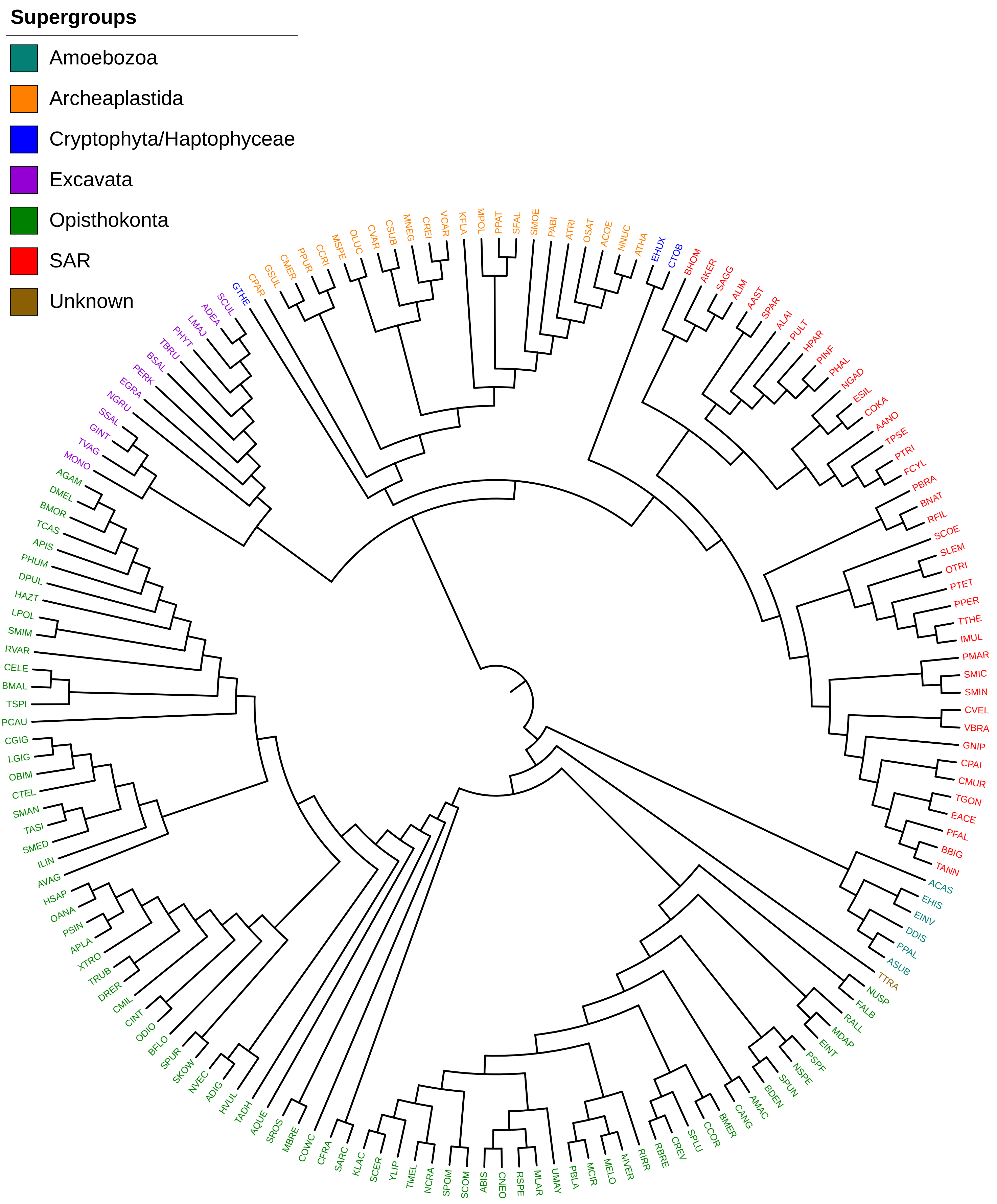

Supplementary Figure 1: Eukaryotic species Tree. The phylogenetic tree of the species used in the analyses and for the Dollo parsimony method. Species names belonging to the ID's in the tree can be found in Supplementary Table 1. The species ID's are coloured according to supergroups and indicated in the legend.

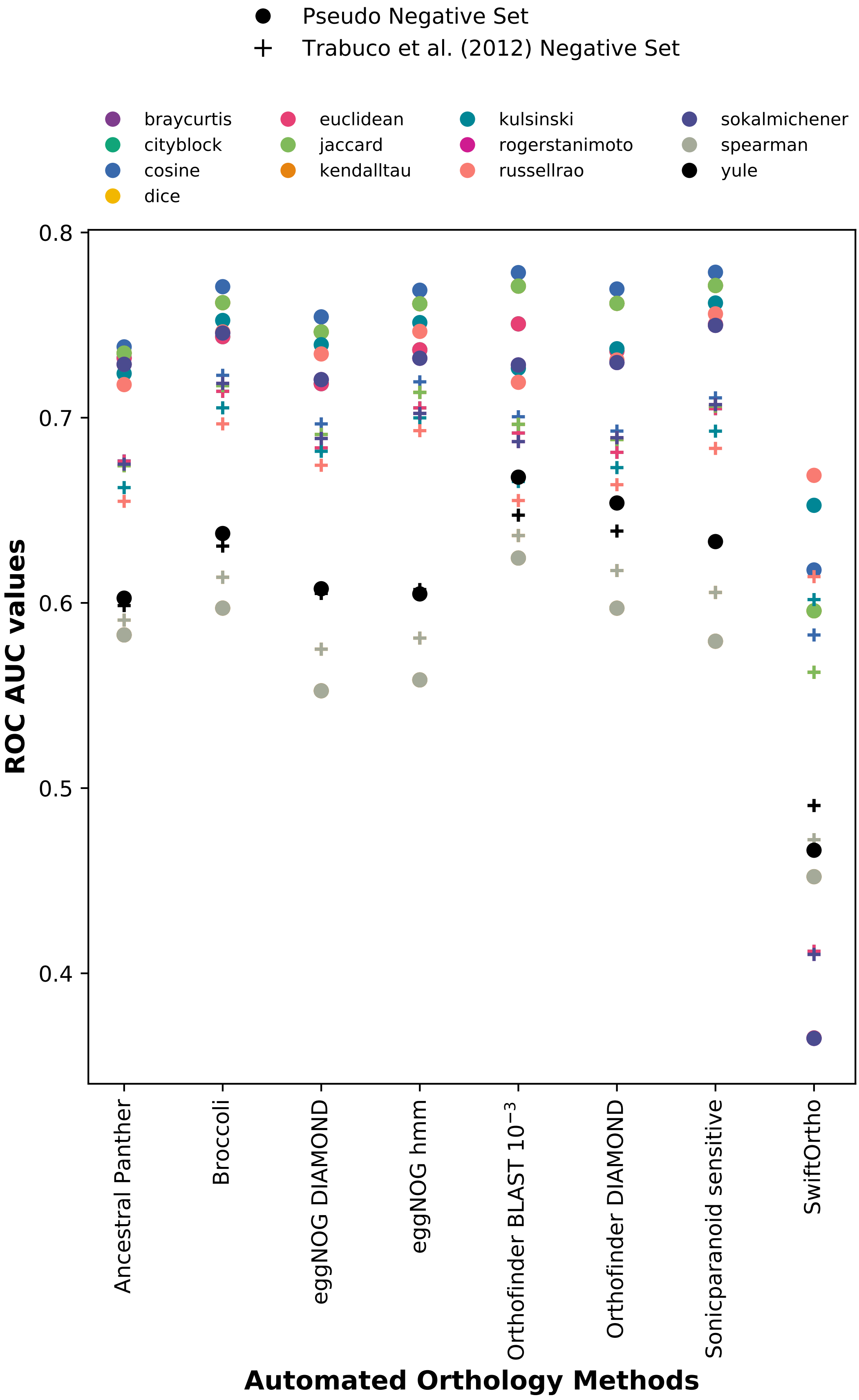

Supplementary Figure 2. Comparison between multiple methods using multiple distances and interaction sets. Calculated ROC Area Under the Curve (AUC) values are shown for all the different methods. Cosine gives the highest AUC value for, except SwiftOrtho, all methods. The Pseudo negative interaction set outperforms the Trabuco et al. (2012) negative interaction set.

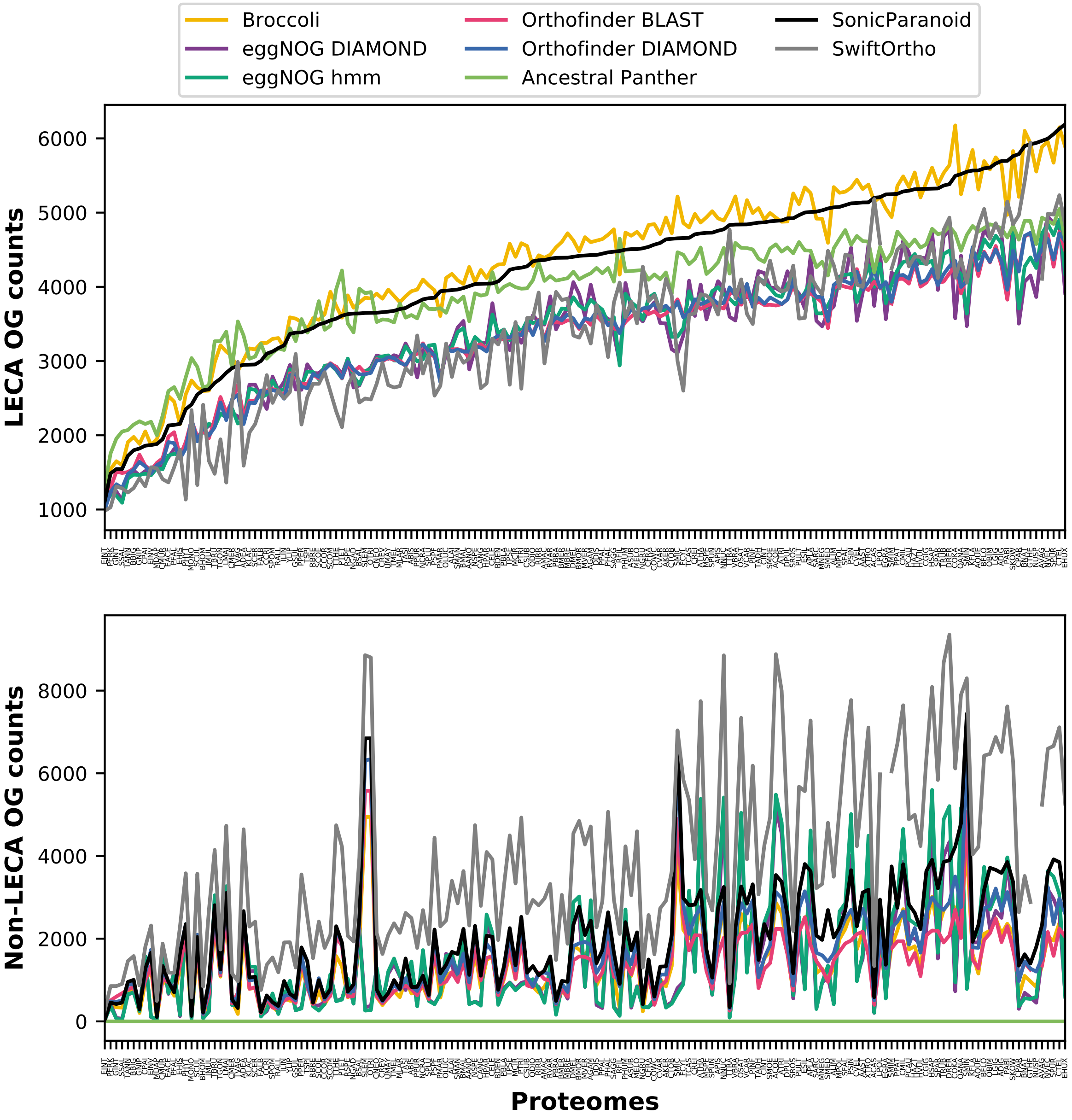

Supplementary Figure 3: LECA OGs and non-LECA OGs per proteome. The values are sorted on the values of SonicParanoid. LECA OGs for every proteome follow similar patterns in all the methods. Note that there are no non-LECA OGs for Ancestral Panther, since these are already ancestral and should in principle only be found in LECA OGs.

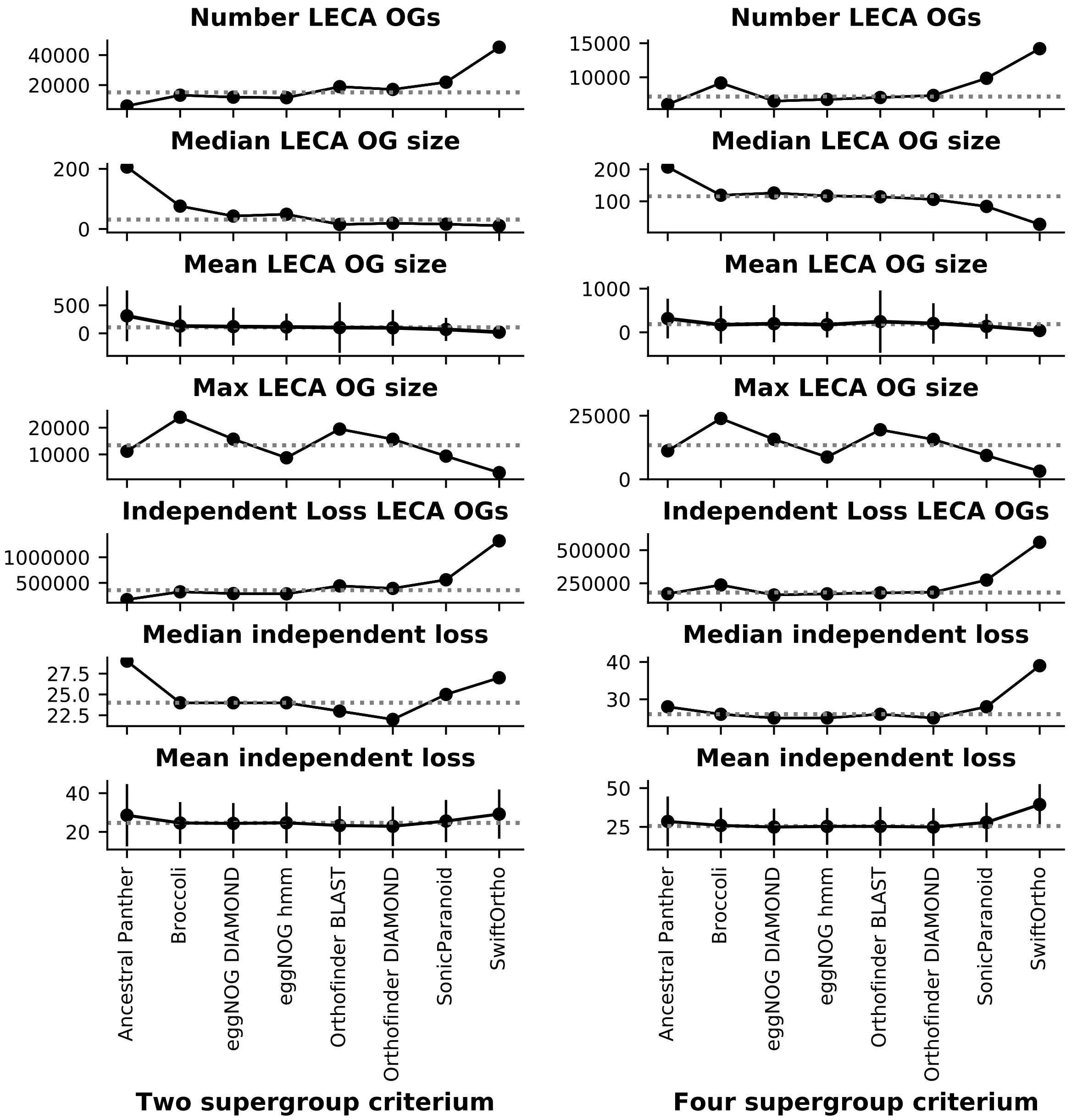

Supplementary Figure 4: Inferred LECA OGs using two and four supergroup criteria. The behavior between the methods is similar to that of the three supergroup criteria.

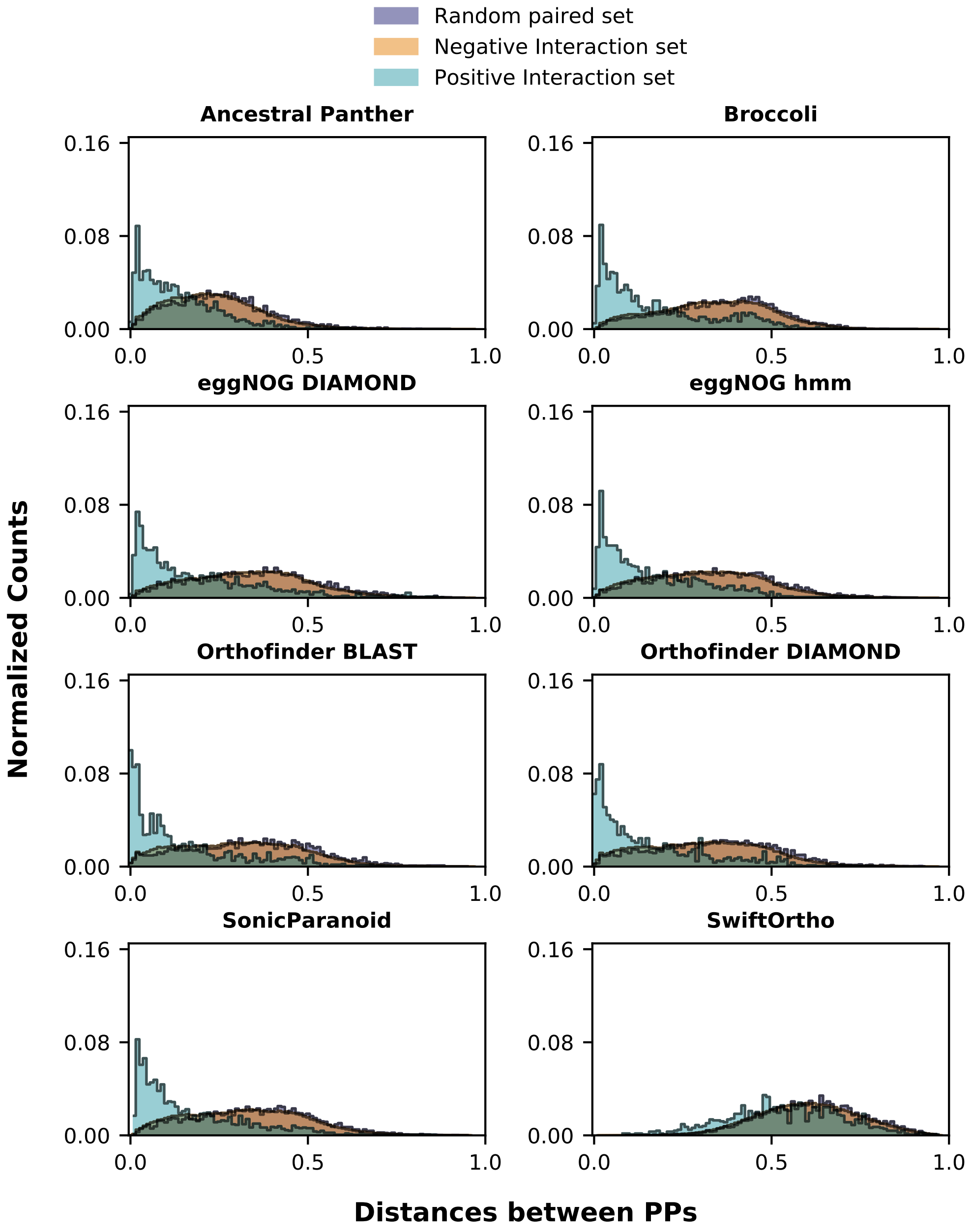

Supplementary Figure 5. Distribution of cosine distances between phylogenetic profiles. Example distributions of the cosine distances between phylogenetic profiles of the positive (blue), negative (orange) and random (purple) protein interaction sets, showing there is a (significantly different) signal in de phylogenetic profile distances for the positive and negative interaction set, compared to the distances of the random protein interaction set (Mann-Whitney U test p-value < 0.001). The counts are normalized by the sum of all counts of the corresponding set.

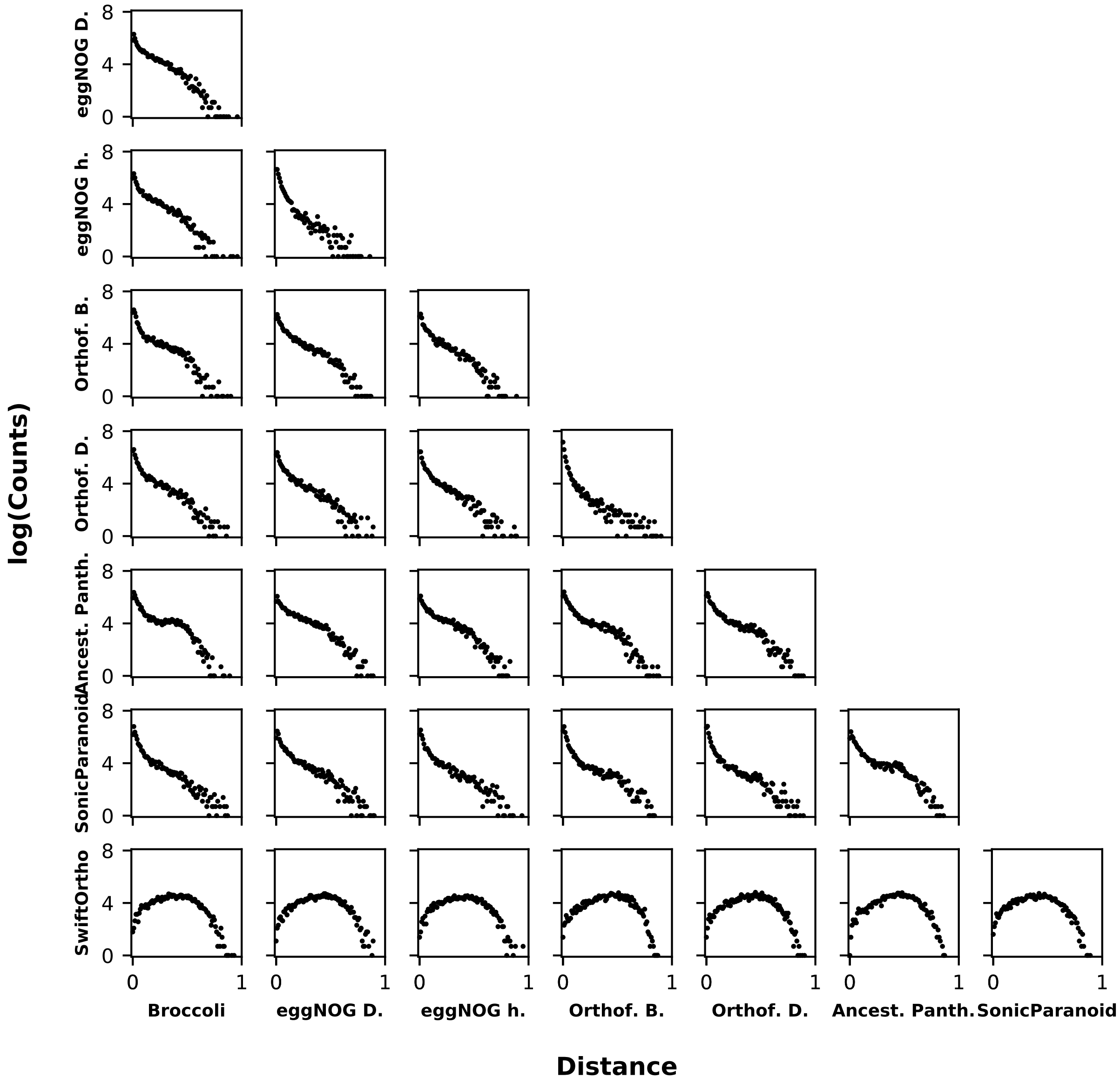

Supplementary Figure 6. The cosine distances between the phylogenetic profiles of OGs from different orthology inference methods. The distances between the phylogenetic profiles of the orthologous groups mapped between the methods should ideally be similar, i.e. a human protein should be found in similar OGs between the different methods. However, this figure shows diversity between the phylogenetic profiles and thus the inferred OGs.

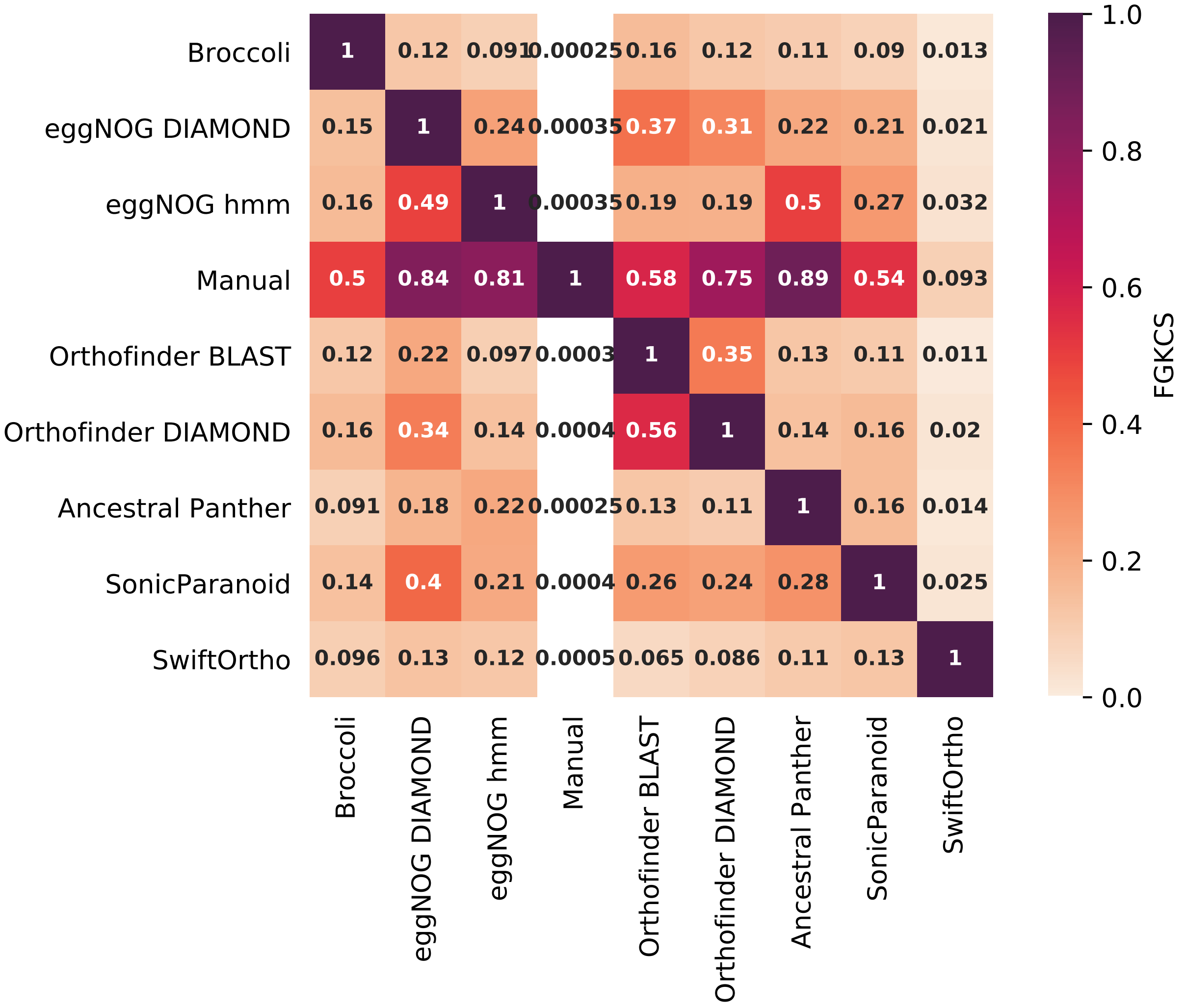

Supplementary Figure 7. The F-Grand K-clique Score (FGKCS) score heatmap. Unlike the ARS (main Figure 4), the FGKCS is not a symmetric score. This way we see how the OGs overlap from method A (vertical axis) to B (horizontal axis), and B to A. For instance, we can make out that the manual OGs (on vertical axis) clusters better with the inferred OGs (on horizontal axis) than the inferred OGs (on vertical axis) cluster to the manual set (on horizontal axis). This has to do with the OG size imbalance between the manual OGs and the inferred OGs (see main text Results).

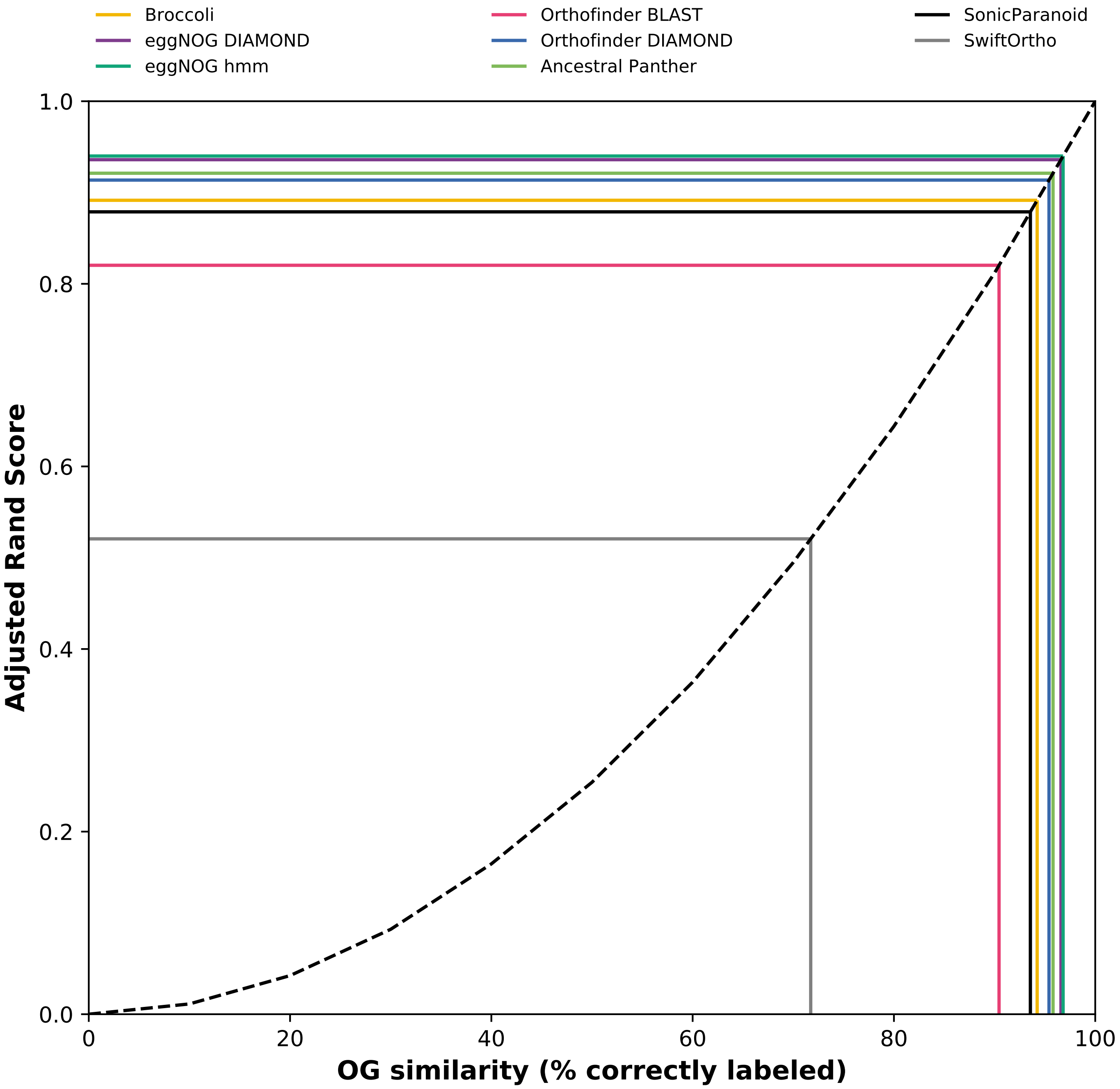

Supplementary Figure 8. The Adjusted Rand Score (ARS) compared to OG similarity. Shows (black dashed line) the relationship of ARS compared to the OG similarity measured as the percentage of unshuffled members (see Methods and Materials main text). The general shape of this relationship is non-linear. The ARS for all the orthology definitions are plotted (colored lines) to approximate the percentage of correctly assigned labels from the manually curated OGs by the automated orthology. The ARS shows a clear difference between the low cluster overlap of SwiftOrtho and the (lower) overlap of Orthofinder BLAST.

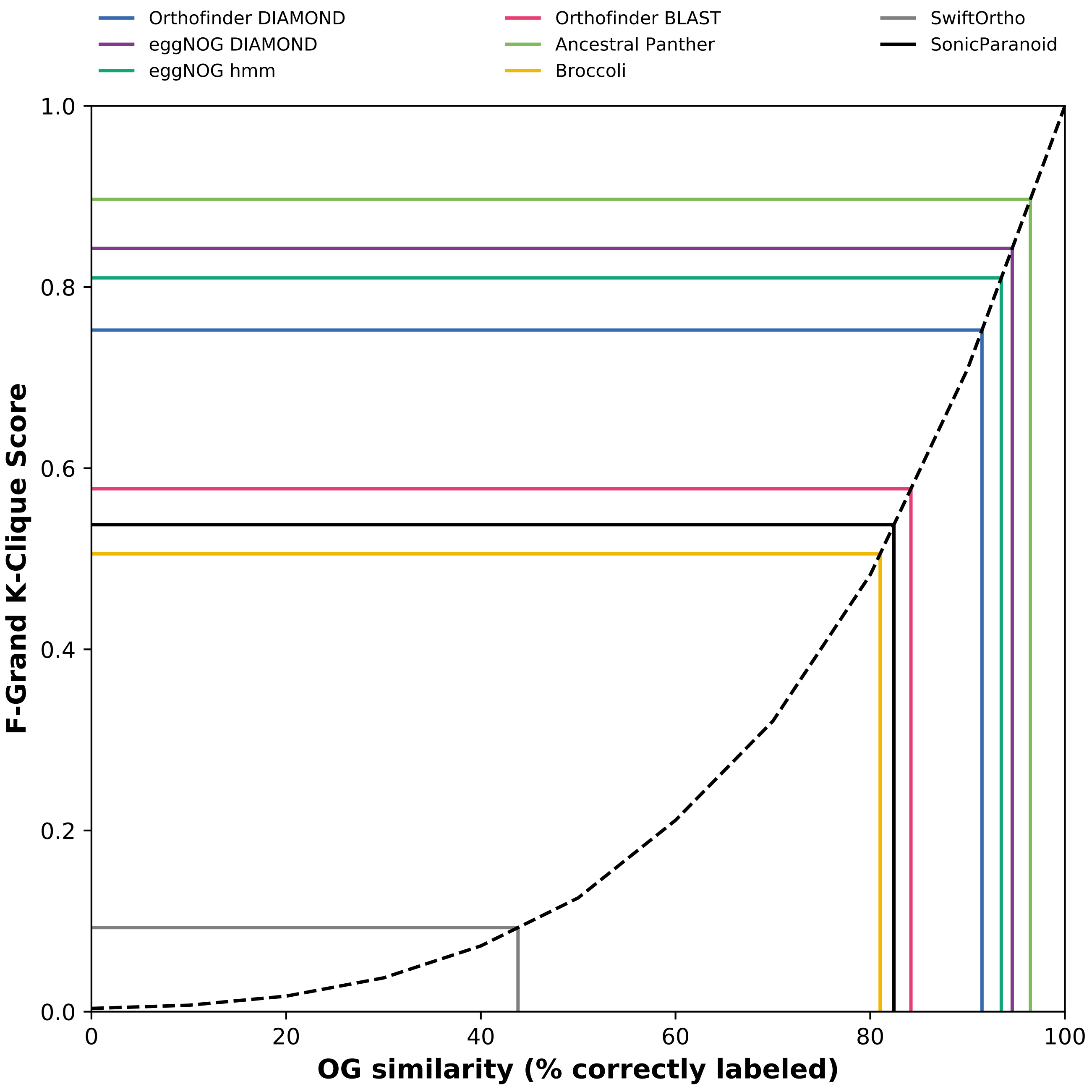

Supplementary Figure 9. The FGKCS compared to OG similarity. Shows (black dashed line) the relationship of FGKCS compared to the OG similarity measured as the percentage of unshuffled members (see Methods and Materials main text). The general shape of this relationship is non-linear. The FGKCS for all the orthology definitions are plotted (colored lines) to approximate the percentage of correctly assigned labels from the manually curated OGs by the automated orthology. The FGKCS shows a clear difference between the (lower) cluster overlap of SwiftOrtho, second tier overlap of Broccoli, SonicParanoid and Orthofinder BLAST and the best cluster overlap for the rest of the orthologies.

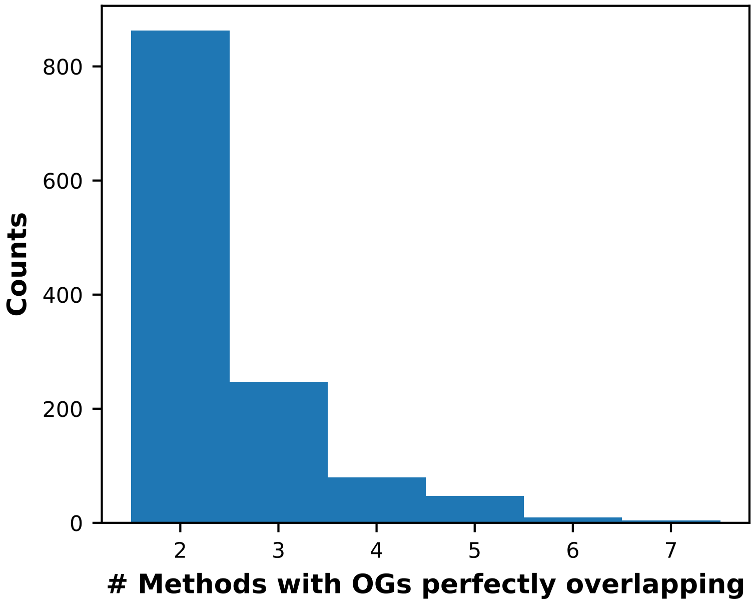

Supplementary Figure 10: Perfectly overlapping LECA OGs between methods. The bulk of the OGs that are identical in what sequences they contain, are so between only two methods. Only four OGs are identical between seven out of eight methods.

Supplementary Figure 11: The fraction of overlap of the clusters between the manually curated OGs and the inferred OGs from different orthology inference methods.

Manual vs. Orthof. DIAMOND

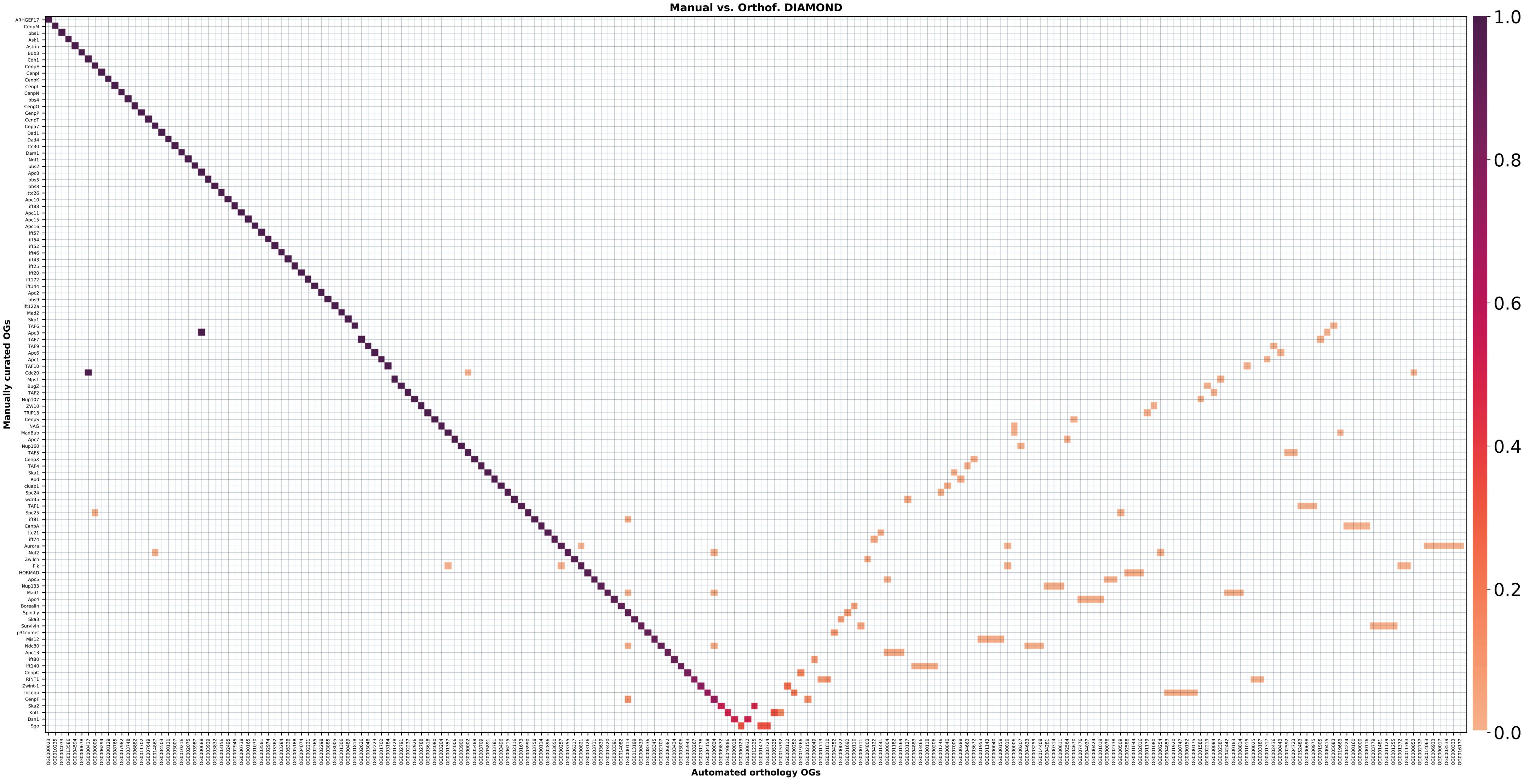

Manual vs. Ancest. Panther

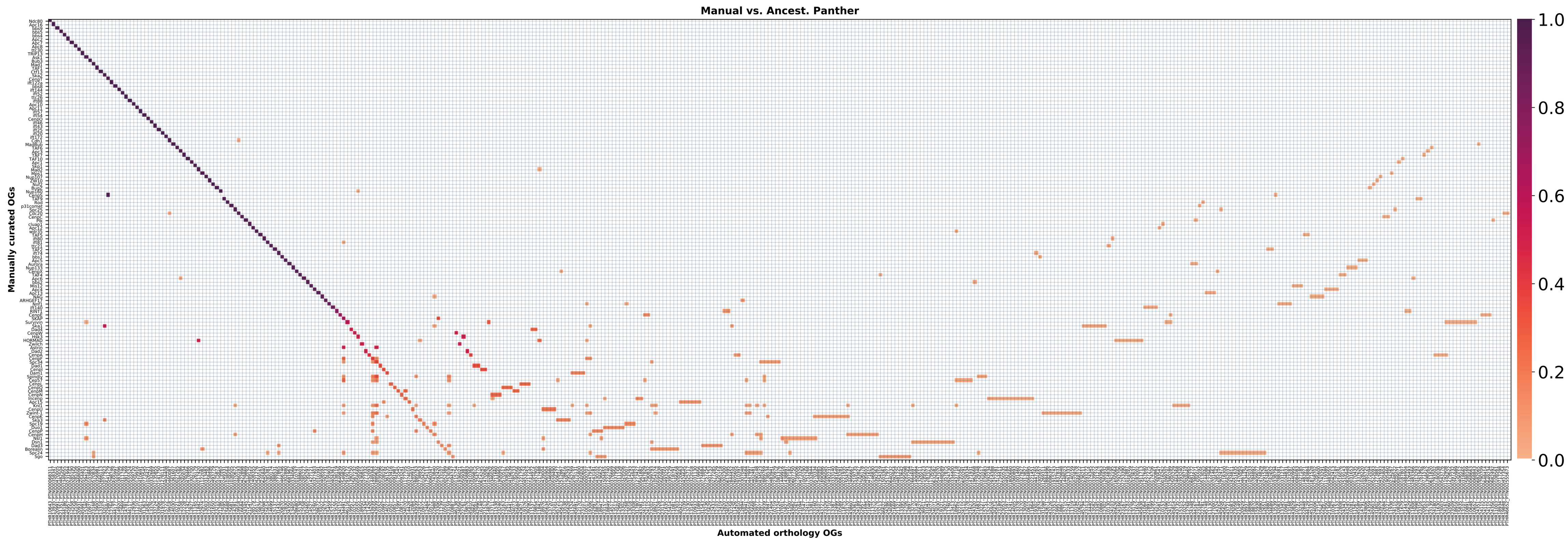

Manual vs. eggNOG DIAMOND

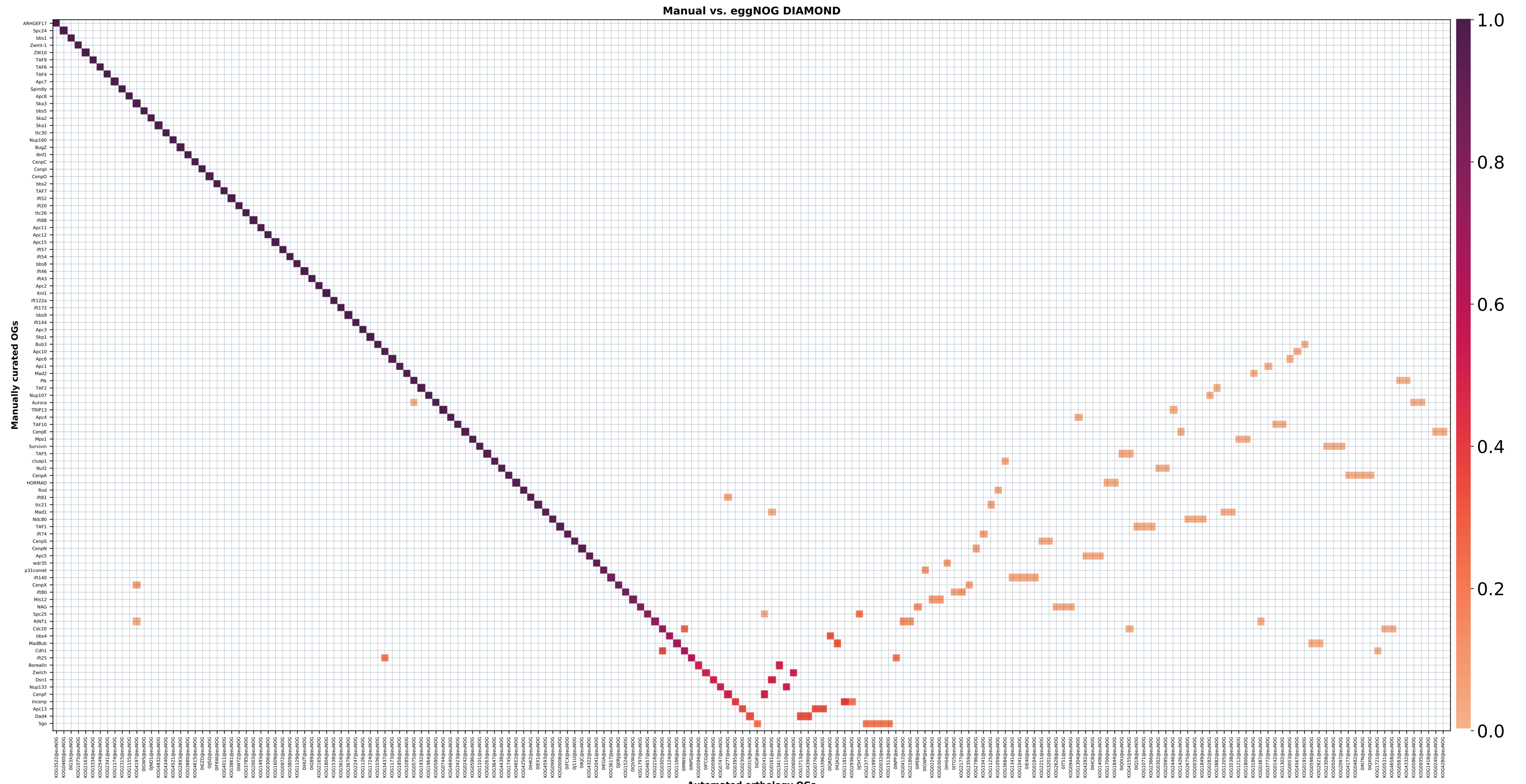

Manual vs. Broccoli

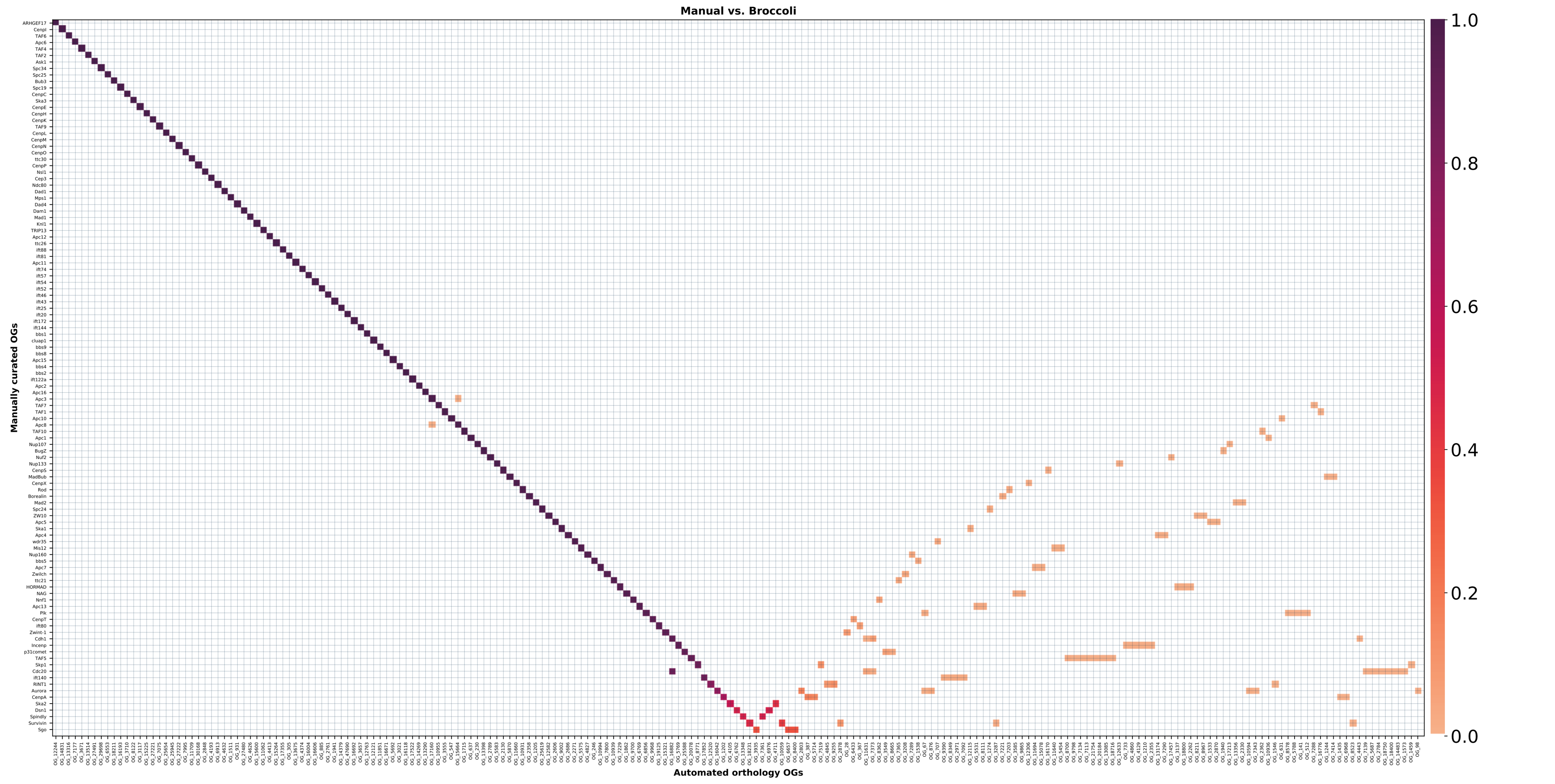

Manual vs. eggNOG hmm

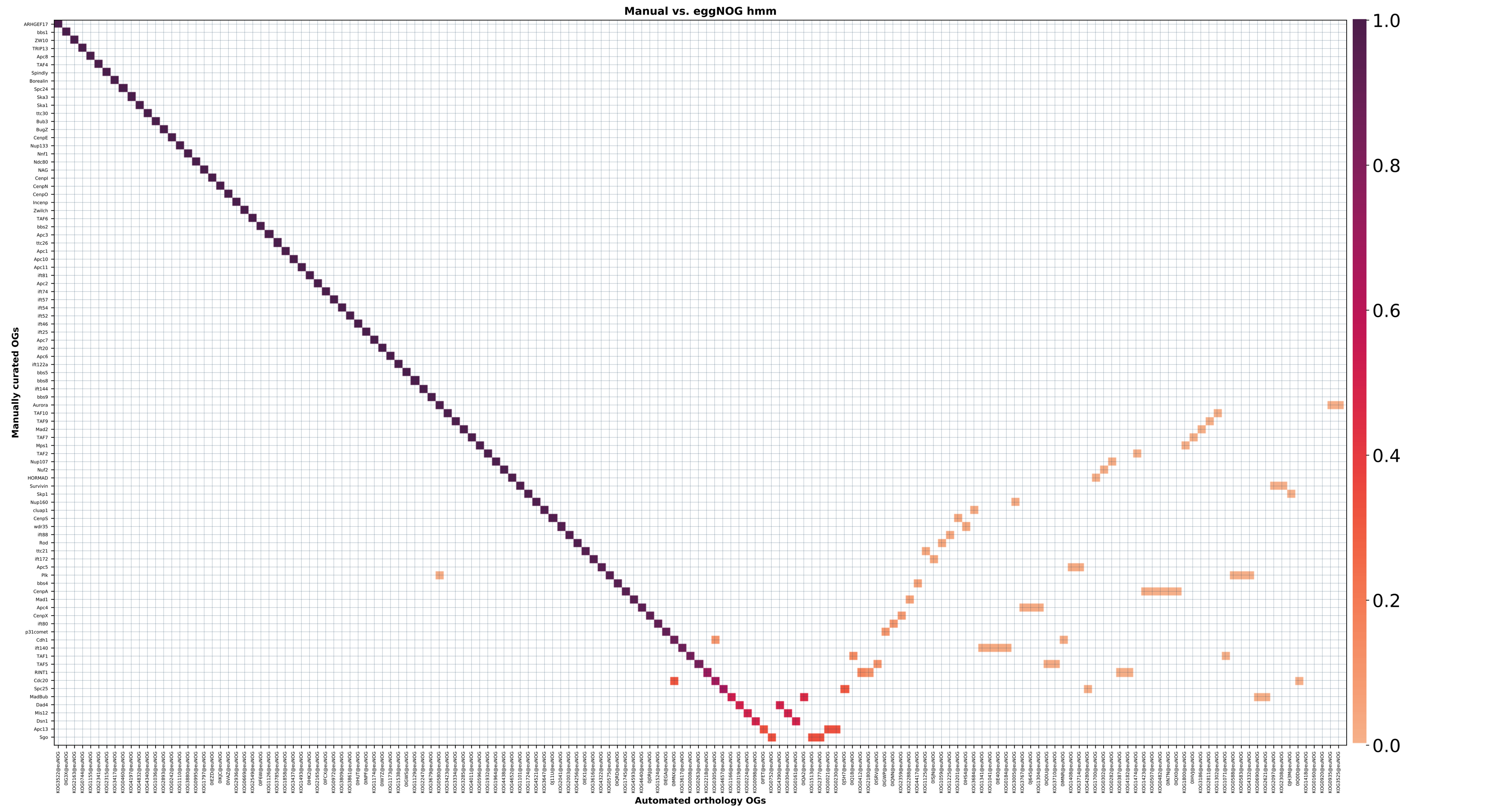

Manual vs. SwiftOrtho

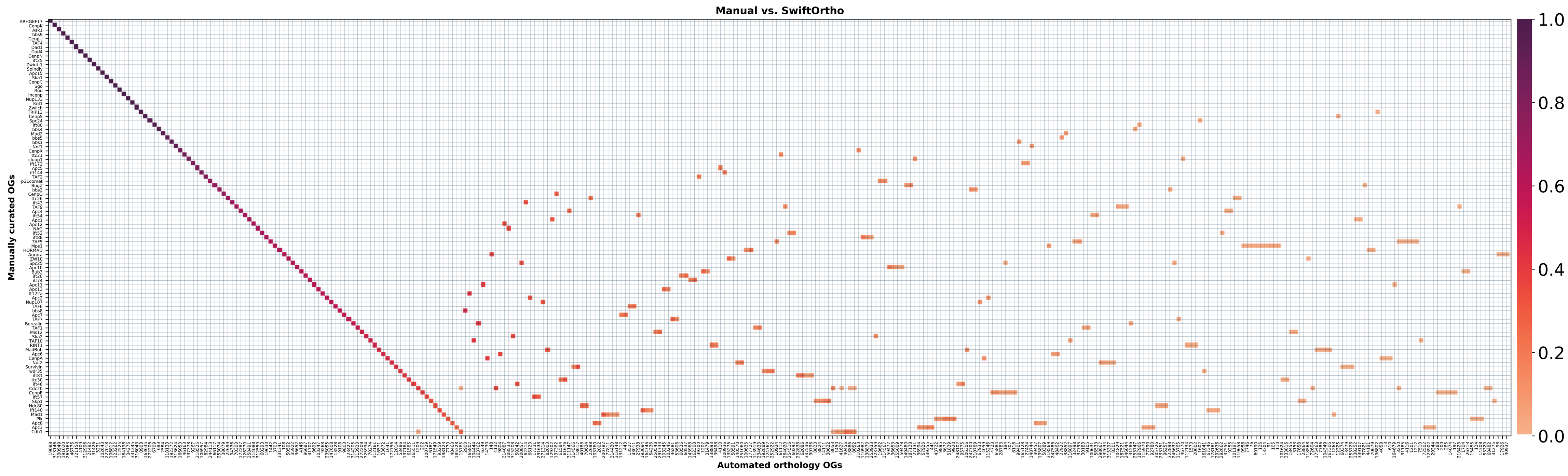

Manual vs. Orthof. BLAST

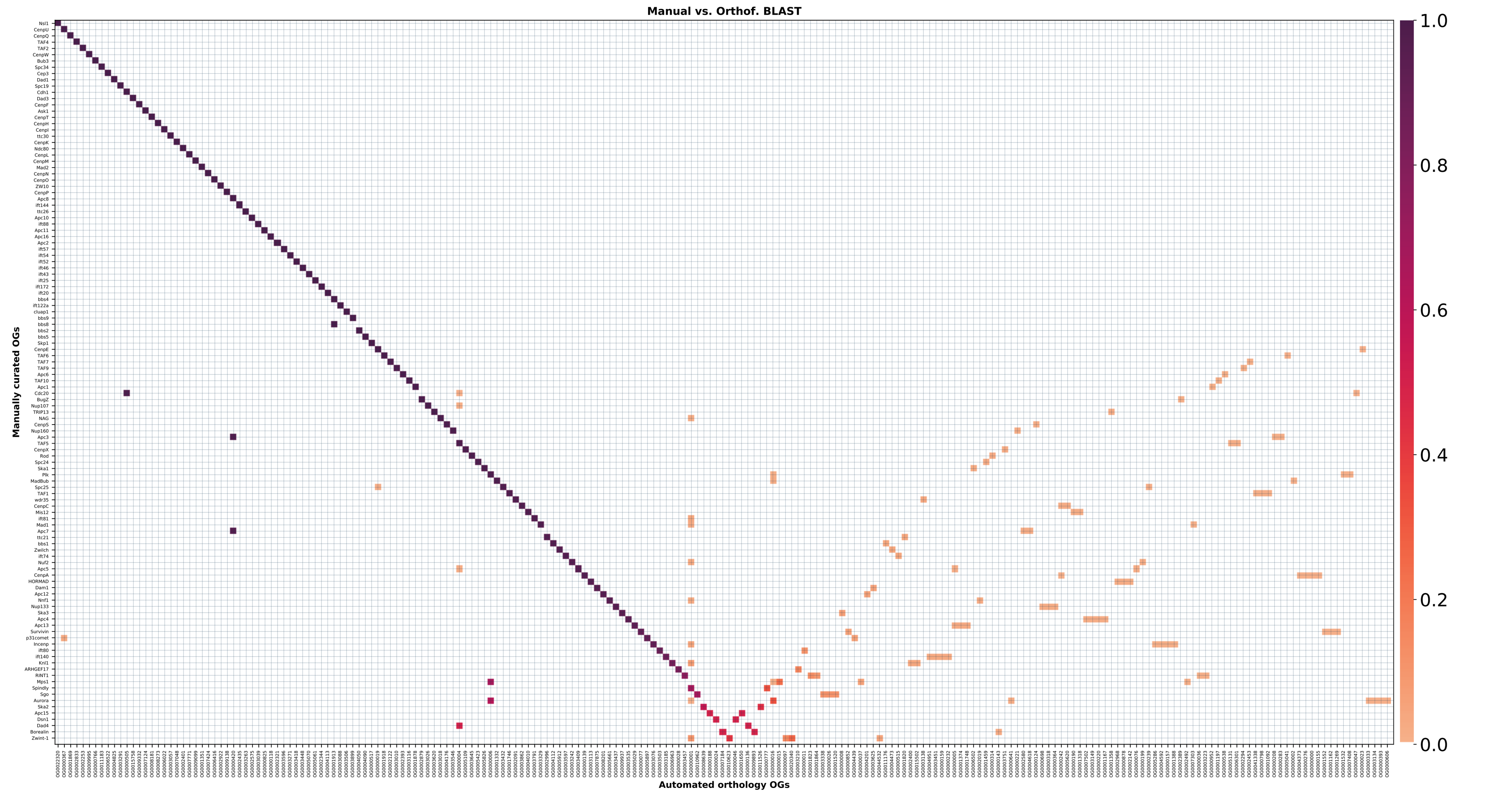

Manual vs. SonicParanoid

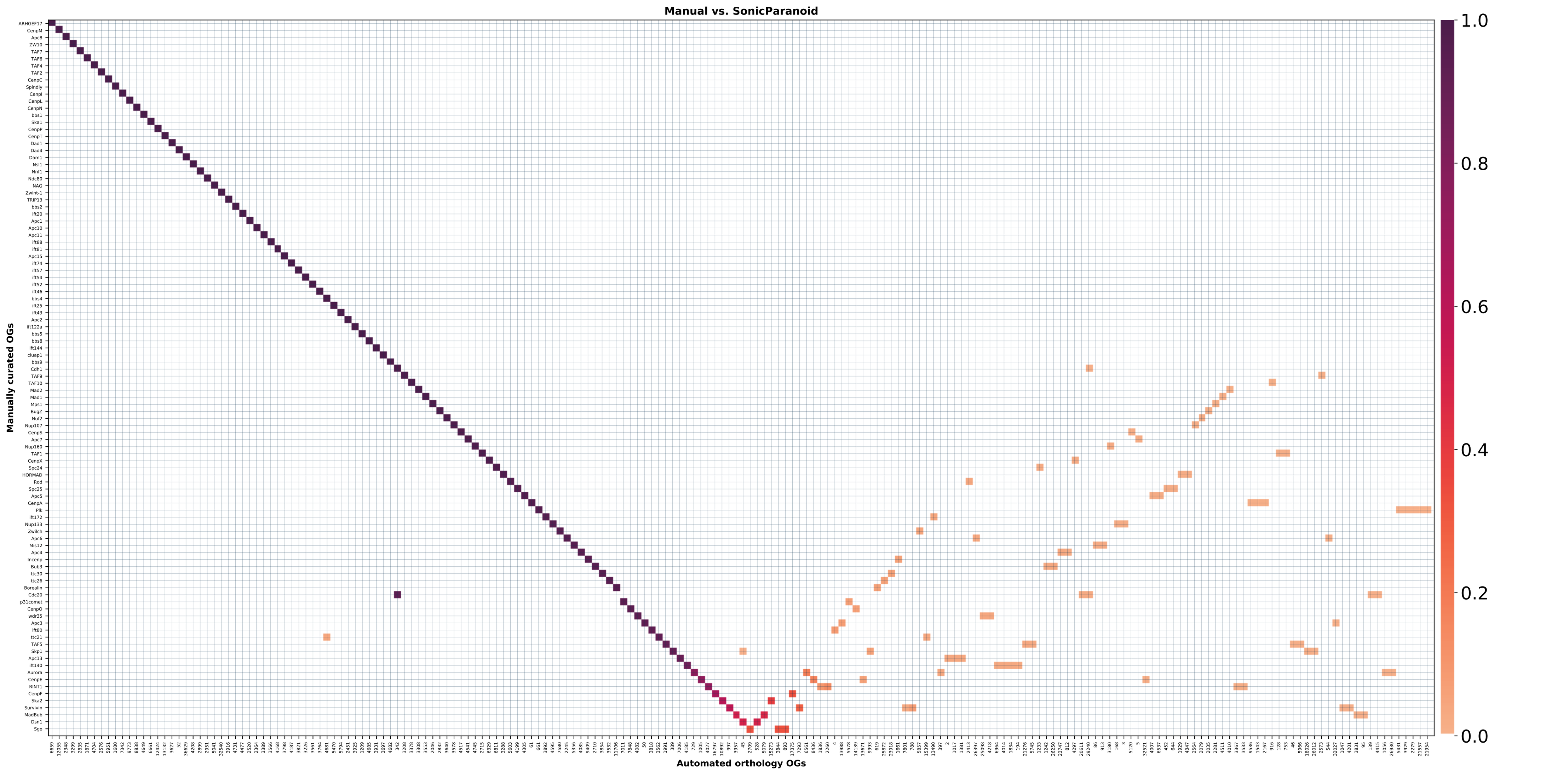

Supplement: Supplementary_Figures_File_bbaa206 [file supplementary_figures_file_bbaa206.pdf]
